# Transcultural Translation and Validation of Lasater Clinical Judgment Rubric©

**DOI:** 10.1590/0034-7167-2021-0880

**Published:** 2022-06-24

**Authors:** Hugo Miguel Santos Duarte, Kathie Lasater, Maria dos Anjos Coelho Rodrigues Dixe

**Affiliations:** I Universidade Católica Portuguesa, Instituto Ciências da Saúde de Lisboa. Lisboa, Portugal.; II Centro Hospitalar de Leiria. Leiria, Portugal.; III Instituto Politécnico de Leiria, Escola Superior de Saúde. Leiria, Portugal.; IV Instituto Politécnico de Leiria, Center for Innovative Care and Health Technology, ciTechCare. Leiria, Portugal.; V Oregon Health & Science University. Portland, Oregon, United States of America.; VI Edinburgh Napier University. Edinburgh, United Kingdom.

**Keywords:** Clinical Judgment, Education, Nursing, Psychometrics, Students, Nursing, Validation Study, Julgamento Clínico, Educação em Enfermagem, Psicometria, Estudantes de Enfermagem, Estudo de Validação, Razonamiento Clínico, Educación en Enfermería, Psicometría, Estudiantes de Enfermería, Estudio de Validación

## Abstract

**Objectives:**

to translate and cross-culturally validate the Lasater Clinical Judgment Rubric© (LCJR©) instrument for nursing students.

**Methods:**

the application of LCJR-PT© was preceded by a linguistic translation into Portuguese, based on the translation-back-translation method. This psychometric study involved 32 nursing students from a program in Portugal. Data were collected through observations of two independent observers during the performance of the practices developed by the students, through the scenarios validated by experts of high and of medium-fidelity simulation.

**Results:**

of the 64 observations obtained from the practices of nursing students, the value of intra-class correlations in the 4 aspects of the instrument exceeded 0.792. There was a global Cronbach’s alpha of LCJR-PT© of 0.921 and 0.876 in Observers 1 and 2 respectively, with a statistically significant level of agreement.

**Conclusions:**

the LCJR-PT© is a valid and reliable instrument, demonstrating a high potential for its use in clinical education and nursing research.

## RESUMO

**Objetivos:**

traduzir e validar transculturalmente o instrumento *Lasater Clinical Judgment Rubric*© (LCJR©) para estudantes de Enfermagem.

**Métodos:**

a aplicação do LCJR-PT© foi precedida por tradução linguística ao português, baseando-se no método de traduçãoretradução. Este estudo psicométrico envolveu 32 estudantes de Enfermagem de um programa em Portugal. Os dados foram recolhidos mediante observações de dois observadores independentes durante o desempenho das práticas desenvolvidas pelos estudantes por meio dos cenários validados por peritos em simulação de alta e média fidelidade.

**Resultados:**

das 64 observações das práticas de estudantes de Enfermagem, o valor das correlações por intraclasse nos quatro aspectos do instrumento ultrapassou os 0,792. Identificou-se um alfa de Cronbach global na LCJR-PT© de 0,921 e 0,876 nos observadores 1 e 2, respectivamente, com um nível de concordância estatisticamente significante.

**Conclusões:**

a LCJR-PT© é um instrumento válido e confiável, demonstrando um alto potencial para seu uso na educação clínica e pesquisa em enfermagem.

## RESUMEN

**Objetivos:**

traducir y validar transculturalmente el instrumento *Lasater Clinical Judgment Rubric*© (LCJR©) para estudiantes de Enfermería.

**Métodos:**

aplicación del LCJR-PT© precedida por traducción lingüística al portugués, basándose en el método de traducción y retraducción. Estudio psicométrico involucró 32 estudiantes de Enfermería de un programa en Portugal. Datos recolectados mediante observaciones de dos observadores independientes durante el desempeño de prácticas desarrolladas por estudiantes mediante los escenarios validados por peritos en simulado de alta y mediana fidelidad.

**Resultados:**

de las 64 observaciones de prácticas de estudiantes de Enfermería, el valor de correlaciones por intraclase en los cuatro aspectos del instrumento sobrepasó 0,792. Identificado un alfa de Cronbach global en el LCJR-PT© de 0,921 y 0,876 en los observadores 1 y 2, respectivamente, con un nivel de concordancia estadísticamente significante.

**Conclusiones:**

el LCJR-PT© es un instrumento válido y confiable, demostrando un alto potencial para su uso en educación clínica e investigación en enfermería.

## INTRODUCTION

Over the past two decades, nursing education has deepened the use of clinical simulation as a learning tool, inserted as an innovative teaching methodology^(1)^. Through High-Fidelity Simulation (HFS), one type of clinical simulation characterized by using technologically advanced, dynamic and computer-controlled mannequins, inserted in a controlled simulation environment, in order to represent real situations in clinical practice, it is possible to consolidate and integrate theoretical knowledge with clinical practice, offering students the possibility of learning and practicing technical skills, prioritizing care skills, delegating interventions, organizing care and teamwork^(2,3,4)^.

In addition, HFS facilitates the learning process, using pedagogical strategies framed in the statute of the centrality of the student that promotes critical thinking, improves clinical reasoning and optimizes clinical judgment in decision making in nursing^(4,5,6,7,8)^.

As it turns out, clinical judgment is a fundamental part of decision making, necessary to carry out an adequate management of the difficulties identified in solving problems^(9 10 11)^. Clinical judgment is a process composed of four aspects – noticing, interpreting, responding, and reflecting – based on the interpretation of the health needs, concerns, or problems that a patient presents, in conjunction with the decision to perform or not intervene, implement, or modify standard interventions or even develop new interventions taking into account the responses obtained in the patient^(12)^.

This process of making clinical judgments is an essential part of the assessment and nursing diagnosis in the planning and implementation of interventions, culminating in the evaluation of these same interventions^(12)^. Effective clinical judgments are essential in nurses and nursing students so that patient safety and quality of care are constant goals in nursing practice, and since their absence is a risk factor for the occurrence of adverse events^(13-14)^.

In nursing care practice, as in the overall practice of health care, it is necessary to consider technical and non-technical skills so that nursing students can learn to diagnose, implement, and evaluate nursing interventions, in a way that is not purely technical. Thus, when problems are analyzed in different ways and perspectives, in the best interest of patients, considering a variety of complex factors for the best choice of the course of action, it can be said that we are dealing with nurses or future nursing professionals with capabilities to make clinical judgments^(12,15)^.

In 2007, an instrument was developed that allows for analysis of the aspects of clinical judgment – the Lasater Clinical Judgment Rubric© (LCJR©)^(15)^. Through this instrument, a common language about clinical judgment is possible so that nursing students and teachers can communicate about, as well as think critically, to allow feedback and discuss the simulation scenarios developed within the scope of medical-surgical nursing^(15)^.

According to the author, the instrument was developed through a mixed methods study, initially through the qualitative analysis of the data referring to 53 observations of nursing students of the second year, within the scope of clinical simulation scenarios in medical-surgical nursing. Subsequently, in quantitative terms, the scores regarding the observations of 26 students during simulation scenarios were analyzed, and finally, a focus group was carried out with 8 of those observed students^(15)^.

The LCJR© is composed of 11 dimensions, emerging from the four aspects of Tanner’s Clinical Judgment Model – noticing, interpreting, responding, and reflecting – the same being outlined by descriptors in each of the 4 levels of development – beginner (1 point), developing (2 points), proficient (3 points) and exemplary (4 points)^(12,15)^. Within the noticing aspect, there are three dimensions: focused observation, recognition of deviations from expected patterns, and information seeking. In the interpreting aspect, two dimensions emerged: prioritize data and make sense of the data. Regarding the responding aspect, four dimensions are highlighted: calm, confident manner; clear communication; well-planned interventions/flexibility; and being skillful. Finally, within the reflecting aspect, there are two dimensions: evaluation/ self-analysis and commitment to improvement^(15)^. Each dimension is scored separately, but students may score up to a total of 44 points^(15-16)^. In the original study with 26 nursing students, the LCJR© showed an average of 23 points (22.98 ± 6.07) and a point variation between 5 and 33 points, but psychometrics related to the construct validity and instrument reliability were not done^(15-16)^.

Since its inception, there have been several LCJR© translations and cross-cultural validations. In 2010, in a quasi-experimental study with 59 nursing students, where the LCJR© was used for assessment, a Cronbach’s alpha of the different dimensions of clinical judgment ranged between 0.810 and 0.884^(17)^. Later studies focused on validity and reliability^(18)^; for example, one using 29 observations scored with the LCJR©, evidenced the following results: inter-class reliability of 0.899; intra-class reliability of 0.908 and a Cronbach’s alpha of 0.974^(19)^. A slightly lower Cronbach’s alpha was identified in cross-cultural validation in Sweden (LCJR-S©), 0.860^(20)^.

In 2015, the Korean version (K-LCJR©) was developed, using the instrument with a sample of 152 nursing students who participated in HFS scenarios and clinical scenarios with a standardized patient. The K-LCJR© instrument was completed by the participants, obtaining an average of 30 points (29.72 ± 5.89) and demonstrating a Cronbach’s alpha per dimension ranging between 0.897 and 0.909, with an overall value of 0.910. Through confirmatory factor analysis, the presence of four factors was evidenced, corresponding to the four aspects of Tanner’s clinical judgment model (☐2 = 39.91; DF = 38; p = 0.385)^(21)^. In this study, the correlations with the highest connection between them (α = 0.01) were also identified, namely between noticing and interpreting (r = 0.970), between noticing and responding (r = 0.980) and between interpreting and responding (r = 0.940)^(21)^.

More recently, the Dutch version (D-LCJR©) was developed, with the evaluation of 52 nursing students in the context of medicalsurgical clinical simulation scenarios, which revealed a Cronbach’s alpha of 0.930, with an intra correlation classes ranging from 0.690 to 0.780. The D-LCJR© instrument had an average content validity index of 85%^(20)^. This instrument was also cross-culturally validated in Spain, in 2018, through 152 observations made in HFS scenarios and medium-fidelity simulation. A Cronbach’s alpha of 0.930 and an intra-class correlation coefficient of 0.930 were obtained^(22)^. A cross-cultural validation for Brazil (LCJR-BV©) also emerged in 179 participants, with a global Cronbach’s alpha of 0.889, and for the aspects of noticing 0.750, interpreting 0.640, responding 0.780 and reflecting 0.630^(23)^.

In 2019, a virtual version (vpLCJR©), of the LCJR© was validated in Sweden within the scope of virtual clinical simulation, using 125 nursing students, where it revealed a global Cronbach’s alpha of 0.931, and for the aspects of noticing 0.907, interpreting 0.860, responding 0.912 and reflecting 0.838^(24)^. In that same year, cross-cultural validation in China (C-LCJR©) appeared, with 157 nursing students, demonstrating a Cronbach’s alpha for the aspects of noticing 0.840, interpreting 0.710, responding 0.810, and reflecting 0.790^(13)^.

In summary, there have been several cross-cultural validations of the LCJR© instrument; however, none of them with psychometric properties evaluated for nursing students in Portugal. Although the Brazilian version of the instrument is the closest to the language, there are terminological differences – words, phrases, and expressions – that demonstrate different meanings between Portuguese in Brazil and Portuguese in Portugal.

## OBJECTIVES

To translate and validate the Lasater Clinical Judgment Rubric©^(15)^ for evaluation of the clinical judgment of nursing students in Portugal (LCJR-PT©).

## METHODS

The methodology applied in this study is now presented.

### Ethical aspects

The LCJR© cross-cultural validation utilized international guidelines, and the rubric author was first asked for authorization to use the instrument, which promptly obtained a positive response^(15)^. The research project also received approval from the Ethics Committee of the Health Sciences Research Unit, of the *Escola Superior de Enfermagem de Coimbra*, and its application was approved by the management of a program in the central region of Portugal.

Once the study was approved, the project and its objectives were presented to potential participants so they could be informed to voluntarily participate. The principles of the Declaration of Helsinki were respected, namely the confidentiality and anonymity of data.

### Study design, period, and location

This psychometric study based in observations (STROBE) was carried out during the period from February to June 2020, at a nursing program in the central region of Portugal. Nursing student practices were observed in a clinical simulation environment, and raters evaluated the clinical judgment of each participant using the LCJR-PT© instrument. The 64 observations, based on what were the interventions performed by nursing students before a victim in cardiopulmonary arrest in need of BLS, in mediumfidelity simulation and HFS scenarios, after validation by experts in clinical simulation and Basic Life Support (BLS), were obtained by two independent observers viewing the 32 participants. The two independent observers were selected for their specific training in clinical simulation and for being experts in decision making.

### Study Protocol – Phase 1 – Translation Process

The LCJR© translation was carried out according to the translation-back-translation method, an internationally supported method consisting of three steps^(25,26,27)^. Firstly, two bilingual people with knowledge in the health field, who speak and write fluently in English, were selected to translate independently the instrument into Portuguese from the original English version. Then, a synthesis of the two versions was carried out, based on the original instrument by one of the authors to create the hybrid version of the Portuguese instrument. The next step was for two other independent people who speak and write fluent English to translate the Portuguese version into the original language of the instrument. The version of the instrument translated into Portuguese was also reviewed by three experts in the field of clinical judgment, who compared it with the original version in terms of terminology and sentence construction, with no linguistic questions. Finally, the author of the original instrument, revised the final version, and adjustments were made after clarification of terminological questions. Using 13 different nursing students from the study sample (volunteers), the instrument items (words, phrases, understanding and interpretation) were terminologically validated. Thus, it was possible to validate each of the items regarding their sentence construction, understanding and interpretation of the Portuguese version of Lasater Clinical Judgment Rubric© (LCJR-PT©).

### Study Protocol – Phase 2 – Validation Study

The sample for this study was 32 second-year nursing students, who volunteered to participate in the study, who had not attended clinical teaching at the time of the clinical simulation scenarios; they had no previous experience in clinical simulation practices; they had not attended clinical practice environments prior to the beginning of the nursing degree course; they had not been trained or certified in BLS, nor had they acted in real situations with BLS.

To recruit these participants, a contact was made with the program involved and a day was scheduled when there were class meetings with the coordination of the nursing course, in a physical presence in the classroom, after approval by the ethics committee and authorization from the School Management and Course Coordination.

Nursing students who participated in this study initially received theoretical training on BLS, followed by division into two groups to participate in clinical simulation scenarios featuring the need for BLS. The clinical simulation scenarios developed by the researchers of this study were validated by experts in the field of clinical simulation and in the field of basic life support. Group 1, within the scope of the HFS, performed the scenario using Resusci Anne Laerdal® simulators, while the group 2 used MegaCode Kelly Laerdal® simulators. The main particularity of HFS scenarios is the fact that their physical fidelity is superior to that of medium-fidelity simulation scenarios, triggering a more natural immersion in a HFS scenario, surrounded by a controlled environment, but with highly developed technological resources. Throughout the HFS scenarios, there was one instructor inside the laboratory and another one inside the control room, while in the medium-fidelity scenarios, there was only one instructor inside the laboratory. Both groups had equal scenarios for the development of interventions in the context of the victim of cardiopulmonary arrest in a hospital environment. All clinical simulation scenarios developed within the laboratories were filmed in audio-visual format with the authorization of the participants. Subsequently, the recordings were analyzed by the two investigators, independently, using the LCJR-PT© instrument. Thus, it was possible to assign scores in each of the dimensions evaluated by the instrument without any interaction between observers and nursing students.

### Analysis of Results and Statistics

For the treatment and analysis of the results obtained, the computer program Statistical Package for the Social Science (SPSS), version 26.0, was used. Descriptive statistical measures were used to characterize sociodemographic and academic data. The validation of LCJR-PT© was based on the successful determination of the psychometric characteristics, and it was necessary to test its reliability and validity^(28)^. Thus, for the LCJR-PT© fidelity analysis, the following premises were analyzed: Cronbach’s alpha coefficient of all the items that make up each instrument, as well as the scale after excluding each of the items individually; through Cronbach’s alpha, it was possible to evaluate the instrument’s internal consistency, which can vary between 0 and 1, with higher values being indicators of better internal consistency; Cronbach’s alpha scores greater than 0.800 demonstrate good internal consistency. The correlation of each item with the total scale was also determined to assess whether each item is a good indicator of the total instrument, if its correlation is greater than 0.200, as well as Kappa’s concordance coefficient to assess the level of agreement between the two observers^(29)^. T-student test was also used to determine differences between two variables. Before the statistical test was employed to identify the relationship between variables, the Shapiro Wilk test was applied to evaluate variable distribution. The significance level used was p≤0.05.

## RESULTS

After completing the translation phase of the instrument, as described above, observations were made of the clinical simulation scenarios developed by nursing students, using two independent observers. Thirty-two nursing students participated in this study, in a sample consisting of 3 (9.4%) male students and 29 (90.6%) female students, with an average age of 19.9 ± 3 years.

With the sample of this study, two different raters evaluated 32 nursing students in the scope of BLS for a total of 64 observations, in an in-hospital environment, to cross-culturally validate the LCJR-PT©. The global mean value of clinical judgment between the two observers was very similar, ranging from 21 to 22 points (observer 1: 22.03 ± 4.748; and observer 2: 21.22 ± 4.202) – see Table 1. Among the eleven dimensions listed by LCJR-PT© it appeared that in both observers, the highest average scores occurred in dimension 8 and 9, respectively *well-planned interventions/flexibility* and *being skillful*. The same was true for the dimension with a lowest average score, corresponding to dimension 4 – *prioritizing data*.

**Table 1:** Characterization of the measurement of clinical judgment by observation and analysis of the intra-class correlation coefficient of the instrument Lasater Clinical Judgment Rubric©

Dimension	Observer 1		Observer 2		t	p	ICC
	M	SD	M	SD			
D-1: Focused observation	1.84	0.677	1.78	0.706	0.701	0.488	0.849
D-2: Recognition of deviations from expected patterns	2.09	0.466	2.03	0.177	0.812	0.423	0.384
D-3: Information seeking	1.97	0.740	1.78	0.751	1.982	0.056	0.841
D-4: Prioritizing data	1.62	0.492	1.75	0.568	-1.277	0.211	0.623
D-5: Making sense of the data	2.16	0.677	2.13	0.793	0.373	0.712	0.888
D-6: Calm, confident manner	1.91	0.689	1.84	0.628	0.701	0.488	0.831
D-7: Clear communication	1.97	0.740	1.94	0.759	0.373	0.712	0.892
D-8: Well-planned interventions/flexibility	2.22	0.420	2.25	0.568	-0.297	0.768	0.458
D-9: Being skillful	2.22	0.420	2.34	0.602	-0.892	0.379	-0.409
D-10: Evaluation/self-analysis	1.88	0.492	1.84	0.448	0.442	0.662	0.783
D-11: Commitment to improvement	2.16	0.369	2.03	0.309	2.104	0.044	0.656
A-1: Noticing	5.90	1.63	5.59	1.45	1.832	0.077	0.885
A-2: Interpreting	3.78	1.00	3.87	1.15	-0.722	0.476	0.872
A-3: Responding	8.31	1.85	7.87	1.56	2.080	0.046	0.851
A-4: Reflecting	4.03	0.739	3.87	0.659	1.539	0.134	0.792
LCJR-PT©	22.03	4.748	21.22	4.202	2.156	0.039	0.934

M – Mean; SD – Standard Deviation; t – Student’s t test; p – Significance; ICC – Intra- class Correlation Coefficient; D – D imension; A – Aspect; LCJR-PT© – Lasater Clinical Judgment Rubric - Por tuguese version©.

Regarding the intra-class correlations of LCJR-PT©, obtained by the analysis of the two observers in the context of clinical simulation scenarios, where the nursing students actively participated, the dimensions that present higher correlational values were the dimensions 5 and 7 respectively, *making sense of the data* and *clear communication*, with values of 0.888 and 0.892. Within each of the aspects that encompass the different dimensions of the LCJR-PT© instrument, it was noted that all intra-class correlation coefficients were greater than 0.792 value obtained in aspect 4 – *reflecting*, with the highest value extracted by the global LCJR-PT©, with 0.934.

Using Table 2, it is evident that the classifications obtained by nursing students are organized into two development level descriptors, specifically *developing* and *proficient*, with *developing* being predominant. Through these same data, the level of agreement among observers also stands out with a statistically significant kappa value greater than 0.803.

**Table 2:** Lasater Clinical Judgment Rubric© levels of nursing students (according to Lasater Clinical Judgment Rubric© and assessment agreement between observers)

Classification	Observer		Observer 2		Kappa	p
	N	%	N	%		
Developing	19	59.4	20	62.5	0.803	0.000
Accomplished	13	40.6	12	37.5		

N – Sample; % – Percentage; p – Significance.

Regarding LCJR-PT© fidelity, Cronbach’s alpha coefficient was calculated for all the items that make up the instrument, as well as the scale after excluding each item one by one, in both observers. Table 3 demonstrates a global Cronbach’s alpha of the instrument of 0.921 and 0.876, in observers 1 and 2 respectively, and the analysis of the correlation of each item with the total scale shows that the minimum value of correlation was 0.561 at Observer 1 and 0.434 at observer 2.

**Table 3:** Corrected item-total correlation analysis and Cronbach’s alpha (excluding item) from Lasater Clinical Judgment Rubric©

Dimension	Observer 1		Observer 2	
	Corrected ItemTotal Correlation	Cronbach’s Alpha (Excluding Item)	Corrected ItemTotal Correlation	Cronbach’s Alpha (Excluding Item)
D-1: Focused observation	0.833	0.905	0.781	0.849
D-2: Recognition of deviations from expected patterns	0.561	0.919	0.434	0.878
D-3: Information seeking	0.790	0.908	0.696	0.857
D-4: Prioritizing data	0.573	0.918	0.620	0.863
D-5: Making sense of data	0.740	0.911	0.617	0.866
D-6: Calm, confident manner	0.787	0.908	0.730	0.854
D-7: Clear communication	0.719	0.913	0.540	0.872
D-8: Well-planned interventions/flexibility	0.696	0.914	0.570	0.866
D-9: Being skillful	0.641	0.916	0.472	0.875
D-10: Evaluation/self-analysis	0.728	0.912	0.704	0.860
D-11: Commitment to improvement	0.592	0.919	0.566	0.870
LCJR-PT© – Cronbach’s alpha	0.921		0.876	

D – Dimension; LCJR-PT© – Lasater Clinical Judgment Rubric - Portuguese version©.

## DISCUSSION

Thirty-two nursing students participated in this study, of which 90.6% were female, with an average age of 20 years. These data compare favorably to the reality of nursing courses in Portugal, in which women represent 81.8% of nursing students^(30)^, as well as the data provided by the *Ordem dos Enfermeiros de Portugal*, which report that the percentage of female nurses in Portugal is 82.2%^(31)^.

Using the data obtained, we noted that the total average scores of nursing students at this level between the two observers were very close, revealing mean values that ranged between 21 and 22 points. These values, according to the classification of the original author, indicate that the participants are at the *developing* stage^(15)^.

In Lasater’s study^(15)^, second-year students were observed in the context of practice in medical-surgical nursing simulation scenarios; the values obtained in the present study were very close to the original study – 23 points.

Taking into account the fidelity of LCJR-PT©, there was a global Cronbach’s alpha of 0.921 and 0.876, in observers 1 and 2 respectively, showing a measurement instrument with good internal consistency^(29)^.

This value of Cronbach’s alpha is in line with the values obtained in other studies: 0.974^(19)^; in Sweden 0.860^(20)^; in Korea 0.910^(21)^; in the Netherlands 0.930^(20)^; in Spain 0.930^(22)^; in Brazil 0.889^(23)^; and in Sweden 0.931^(24)^.

Regarding the correlation of each item with the LCJR-PT©, a minimum correlation value of 0.561 was observed in observer 1 and 0.434 in observer 2, thus indicating that it has acceptable validity^(29)^.

The present study involved 64 observations, a number slightly higher than that obtained in other studies – 26 to 59 observations^(15,17,19-20)^, although less than other studies – 152 to 179^(13,21,22,23,24)^.

Regarding the intra-class correlations of the LCJR-PT©, although all of them were greater than 0.792, dimensions 5 and 7 are shown, respectively *making sense of the data* and *clear communication*, with values of 0.888 and 0.892, for a global intra-class correlation of 0.934. These data, indicators of a good level of agreement between the two observers^(29)^, also appeared in a similar direction as in previous studies: 0.908^(19)^; between 0.690 and 0.780^(20)^; and 0.930^(22)^.

### Study strengths and limitations

The strength of this study focused on the cross-cultural validation process of the LCJR© into Portuguese of Portugal, in accordance with international scholarly guidelines. Another strength is related to the fact that this instrument validation is part of a research program on the effectiveness of decision-making by nursing students in high-fidelity clinical simulation, given that clinical judgment is the fundamental pillar of an adequate management of difficulties for the optimization of decision-making in nursing^(9,10,11)^. In this study, one of the limitations was found in the convenience sampling implemented for data collection^(32)^. The second limitation found in this study is related to the sample size. We suggest, in future research work, for the validation of measurement instruments, the increase in the number of students under study, in order to carry out a robust factor analysis. The third limitation is related to the selection of participants from only one program who have not yet experienced clinical practice to develop their clinical judgment.

### Contributions to Nursing

This research allowed us to translate and culturally validate the LCJR-PT© into Portuguese, opening the door to the investigation of clinical judgment in nursing students in Portugal. Now that there is an instrument that allows the evaluation of aspects of clinical judgment, programs will be able to refine strategies for its improvement. Further studies with other Portuguese nursing programs are needed.

## CONCLUSIONS

The LCJR-PT© translation and back-translation process for cultural validation involved independent bilingual translators, participants different from the study sample (13 volunteer nursing students) and experts in the field of clinical judgment. The main objective of this study was to translate and validate the LCJR© measurement instrument for Portuguese nursing students – LCJR-PT©, and involved the participation of 32 nursing students, mostly female, in their second year of the nursing course. LCJR-PT© revealed a global Cronbach’s alpha of 0.921 and 0.876, in observers 1 and 2 respectively, showing an instrument with good internal consistency. It consists of 11 dimensions, distributed over the four aspects of Tanner’s clinical judgment model – noticing, interpreting, responding, and reflecting – corresponding to four levels of development – beginning, developing, accomplished and exemplary – as in the original instrument. The study demonstrated that LCJR-PT© has adequate validity and reliability to evaluate the four aspects of clinical judgment in Portuguese nursing students.

For future studies, a more representative sample of Portuguese nursing students is suggested, with the involvement of participants from several programs, in greater numbers and at different levels, including those with clinical practice experience.
